# Primary adenocarcinoma of the seminal vesicle

**DOI:** 10.4103/0970-1591.45557

**Published:** 2009

**Authors:** M. Tarján, I. Ottlecz, T. Tot

**Affiliations:** Department of Pathology and Clinical Cytology, Central Hospital, Falun, Sweden; 1 Department of Urology Toldy Ferenc Hospital, Cegléd, Hungary

**Keywords:** Adenocarcinoma, differential diagnosis, seminal vesicle

## Abstract

Primary adenocarcinomas of the seminal vesicle (SVC) are very rare and poorly understood neoplasms with only somewhat more than 50 histologically confirmed cases reported in the literature. We demonstrate a case of SVC and discuss the problems related to diagnosis in this tumor.

## INTRODUCTION

Primary adenocarcinomas of the seminal vesicle (SVC) are very rare and poorly understood neoplasms with only somewhat more than 50 histologically confirmed cases reported in the literature.[1] We demonstrate a case of SVC and discuss the problems related to diagnosis in this tumor.

## CASE REPORT

A 54-year-old man with dysuria and haematuria was first examined in January 2007. Laboratory parameters including serum prostate specific antigen (PSA) level were normal. Digital rectal examination detected a poorly defined hard tumoral mass on the site of the left seminal vesicle, under the intact rectal mucosa. This finding was confirmed by ultrasonography. Primary rectal tumor was ruled out by colonoscopy. Microscopic analysis of the colon biopsy showed infiltration of poorly differentiated adenocarcinoma restricted to submucosa with no evidence of tumor or dysplastic change in the mucosa.

**Figure 1:**
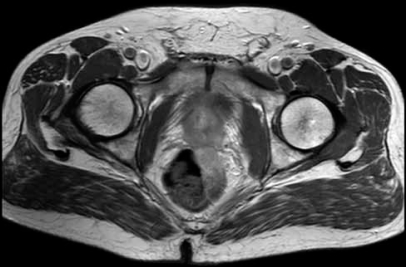
Contrast-enhanced magnetic resonance image demonstrating a tumor mass in the area of the left seminal vesicle, slightly affecting the prostate and the anterior wall of rectum

Contrast-enhanced magnetic resonance imaging was performed, which demonstrated a tumor mass in the area of left seminal vesicle, partly affecting the prostate and the anterior wall of the rectum [Figure 1]. The patient underwent transrectal core biopsy, which histologically showed desmoplastic stroma and fatty tissue infiltrated by the structures of poorly differentiated adenocarcinoma in close relation to the structures of a normal seminal vesicle [Figure 2]. Immunohistochemical analysis evidenced positive reactions with carcinoembryonic antigen (CEA) and cytokeratin 7 in almost all tumor cells, but negative reactions with PSA, CDX2, cytokeratin 20, CA-125 and androgen receptors.

**Figure 2A:**
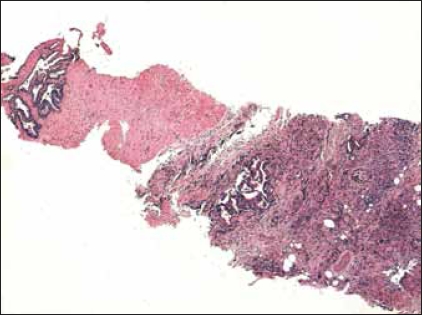
Low magnification histology image of the poorly differentiated tumor infiltrating the surrounding tissue of seminal vesicle (H&E, ×40)

**Figure 2B:**
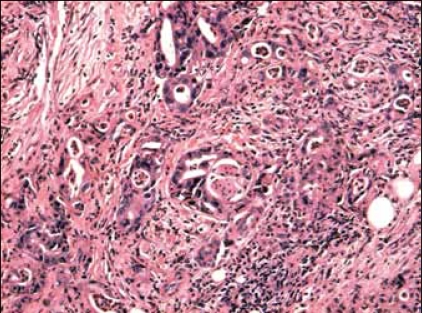
Reprepresentative high-power histology image showing poorly differentiated adenocarinoma with desmoplastic stroma (H&E, ×100)

Chest X ray and computed tomography (CT) of abdomen were also performed without showing any signs of malignancy. No previous diagnosis of malignant disease was registered in the patient's files. The clinical, radiological, histological, and immunohistological findings were consistent with primary SVC. The expert team of urological oncologists proposed radiotherapy before surgical intervention. However, the patient died of the disease 12 months after the histological diagnosis. Autopsy was not performed because relatives requested it.

## DISCUSSION

In 1956, Dalgaard and Giertson[2] established the following criteria for a diagnosis of SVC:

- The tumor should be a microscopically verified carcinoma, localized exclusively or mainly to the seminal vesicle.
- The presence of other simultaneous primary carcinoma should be excluded.
- The tumor should preferably resemble the architecture of the non-neoplastic seminal vesicle.

These criteria can be readily applied to surgical resection specimens, but are less suitable in evaluating radiologically guided needle biopsies. Immunostains are helpful in these circumstances but vesicula-specific markers are not available.[3]

While primary SVCs are rare, secondary neoplastic involvement of the seminal vesicle is not so infrequent. Moreover, when clinical symptoms occur in a patient with SVC, they often reflect advanced disease with infiltration of adjacent organs obscuring the vesicular origin of the tumor. The most common symptoms are non-specific. These are hematuria and hematospermia, but dysuria; painful defecation and general pelvic pain have also been reported.[1] CT scan, magnetic resonance imaging and ultrasonography may be helpful in localizing the tumor. In early stage of the disease these radiologic examinations can help to exclude carcinoma of the adjacent organs, like prostate, rectum or urinary bladder. The modern radiological examinations might also demonstrate the epicentre of tumor in advanced stage of disease.

Histological examination is central in diagnosing SVC, despite the lack of organ-specific immunofenotype. Immunohistochemistry is rather helpful to exclude some metastatic tumors than to confirm the origin of the tumor in seminal vesicle [Table 1]. The common histological variant of SVC is well-differentiated adenocarcinoma with papillary structures[3,4] but poorly differentiated carcinomas similar to our case have also been described.[3,5] The related literature is concordant in the fact that SVC is negative for PSA and also for anti-prostate specific acid phosphatase (PSAP). As the seminal vesicles are often invaded by prostatic adenocarcinoma, the lack of PSA immunostaining in SVC is very useful differential diagnostic clue, but does not completely exclude anaplastic prostatic adenocarcinoma, which may be PSA negative.[5] SVC usually expresses CK7 but not CK 20. Importantly, the rare non-enteric type of bladder adenocarcinoma and adenocarcinoma arising in müllerian duct cyst are also CK7 positive and CK20 negative. In addition, ductal-type of prostatic cancers is PSA positive but usually these tumors are CK7 and CK20 negative. The lack of CK20 immunoreactivity in SVC is helpful in distinguishing these tumors from colorectal adenocarcinoma and urothelial-type bladder carcinoma, usually expressing CK20.

**Table 1:** Immunohistochemical profile of seminal vesicle adenocarcinoma compared to the profile of the tumors representing the most common differential diagnostic options

	Cytokeratin7	Cytokeratin20	CA-125	CEA	PSA	CDX2
Seminal vesicle cancer	+	−	+*	+/−	−	−
Prostate cancer	−**	−	−	−	+	−
Rectal cancer	−	+	−	+	−	+
Urinary bladder adenocarcinoma	+°	−°°	−/+	−	−°°
Müllerian duct adenocarcinoma	+	−	+/−	+/−	−	−

* Poorly differentiated tumors are negative

** ductal type prostate cancers may be positive

° enteric types urinary bladder adenocarcinomas are negative

°° enteric types urinary bladder adenocarcinomas are positive

According to the reported data in the literature, the immunohistohemical profile of SVC is not as consistent regarding other tumor markers as for PSA, CK7, and CK20.[3,5] Carcinoembryonic antigen (CEA) was expressed in most reported cases of SVC, including our case.[1,5] Most well-differentiated papillary SVCs are CA-125 positive, while poorly differentiated SVCs, like our case, do not express this marker of müllerian duct differentiation.[3,5] Thus müllerian duct cyst adenocarcinoma originated in seminal vesicle may be indistinguishable from conventional SVC. The most useful antibodies and their expression in tumors representing the differential diagnostic options are summarized in Table 1.

In summary, the diagnosis of SVC is based on correlation of clinical, radiological, and histological findings. Immunohistochemistry may be helpful in ruling out some differential diagnostic options but cannot per se distinguish poorly differentiated SVC from metastatic spread of poorly differentiated carcinoma of prostate, certain adenocarcinomas of urinary bladder and from the rare carcinomas originating in müllerian duct cyst.
